# Research on Precise Detection Methods for the Maturity of *Pleurotus ostreatus* in Complex Mushroom Cultivation Environments

**DOI:** 10.3390/s26092583

**Published:** 2026-04-22

**Authors:** Jun Yu, Changshou Luo, Qingfeng Wei, Yang Lu, Yaming Zheng

**Affiliations:** 1 Institute of Data Science and Agricultural Economics, Beijing Academy of Agriculture and Forestry Sciences, Beijing 100097, China; yujun2729@163.com (J.Y.); weiqingfeng201@163.com (Q.W.); lysunshineland@126.com (Y.L.); zhengyaming@baafs.net.cn (Y.Z.); 2 Key Laboratory of Intelligent Seedling Technology Innovation, Ministry of Agriculture and Rural Affairs, Beijing 100097, China; 3 Beijing Engineering Research Center of Rural Remote Information Services, Beijing 100097, China; 4 Beijing Key Laboratory of Crop Molecular Design and Intelligent Breeding, Beijing 100097, China

**Keywords:** HSV hue range, 4th channel, physiological prior, YOLOv13, dual-path, hypergraph, StockenAttention

## Abstract

**Highlights:**

A. Physiologically Inspired Color Prior Constraint Based on Physiology

Innovative mechanism: Transformed the physiological color evolution law of *Pleurotus ostreatus* (HSV color distribution) into the fourth-channel input of the network (dynamic color mask) and spatial channel attention weights.

Environmental adaptation: For the first time, LED environment-induced hue transitions ([110°, 155°]) were discovered and utilized, solving the problem of color drift under different lighting conditions.

B. Structured Feature Enhancement

Hypergraph topology modeling: Improved the hyperedge generation mechanism by embedding color priors as node attributes, achieving feature aggregation of “semantic consistency of same color regions”, significantly enhancing the recognition ability of small targets (such as *Pleurotus ostreatus*).

Lightweight design: Significantly improved the semantic expression ability of the model while maintaining its lightweight (only +10.5% parameters) and high real-time performance (>12 FPS).

**What are the main findings?**

- Discovery of environmental color physical model.

Core finding: Under LED illumination, *Pleurotus ostreatus* undergo a color transition to the blue-green system ([110°, 155°]) due to chlorophyll fluorescence, providing a physiological basis for visual detection based on light.

- Effective injection of color priors.

Technological innovation: By using the fourth-channel color mask and ColorWeight module, the color evolution law is embedded into the network, significantly improving the accuracy of maturity discrimination (accuracy increased by 6.1–7.11%) and reducing the misjudgment rate by 73.6%.

- Feature aggregation for hypergraph modeling.

Breakthrough difficulty: The improved hypergraph relationship modeling solves the problem of insufficient feature capture of small targets (such as *Pleurotus ostreatus*) by CNN, significantly improving small target detection.

- Lightweight industrial implementation.

Application value: The model is lightweight and efficient (with only +10.5% parameters), achieving a 13 FPS inference speed on a regular PC server and achieving an F1 score of 0.91 in a smart mushroom room, with a missed detection rate of only 1.2%.

**What are the implications of the main findings?**

- The significance of smart agriculture.

Proposed a universal “environment feature model” linkage scheme to provide reference for color feature extraction of multiple crops.

- The significance of computer vision.

Verified the efficient role of structured priors (color, shape knowledge) in complex backgrounds.

- Research implications.

Suggested introducing thermal imaging/depth information for multimodal fusion and exploring dynamic lighting control based on model output.

**Abstract:**

Addressing the challenges of complex background interference, low lighting conditions, small target recognition, and difficulty in maturity grading in the automated detection of *Pleurotus ostreatus*, this study proposes a lightweight improved scheme based on color feature enhancement. By collecting 4779 images from five developmental stages in three typical planting environments, including greenhouses and mushroom houses, an HSV hue analysis database was established to determine key hue intervals [4°, 38°] or [110°, 155°] for different environments. Secondly, based on the hue interval distribution of *Pleu-rotus ostreatus*, YOLOv13 was used as the base model, with the addition of an HSV hue mask as the fourth channel to improve the input layer. The custom ColorWeight module was used to enhance color feature expression; the hypergraph computation module was improved to enhance feature correlation; and the neck network incorporated the StockenAttention module to improve the ability to capture maturity features. The accuracy of the improved model was increased to 89.5% in mAP@0.5 (+3.3%), surpassing the mainstream YOLOv8n-12n series. Efficiency optimization achieved real-time detection at 12.58 FPS on the RTX3090Ti platform. In practical applications, the accuracy of maturity recognition was significantly improved, with a 73.6% decrease in the misclassification rate of maturity and a reduction in missed detections, achieving an F1 score of 0.91. In conclusion, through the deep integration of Hue features and deep learning models, while ensuring lightweight deployment (with only a 10.5% increase in parameter count), the accuracy and practicality of *Pleurotus ostreatus* detection were significantly improved, providing an effective solution for intelligent mushroom house management.

## 1. Introduction

*Pleurotus ostreatus* is one of the edible fungi with the highest yield and economic benefits at present. Its maturity not only determines taste and flavor, but also is directly related to its market value and safety. With the continuous improvement of consumers’ demand for high-quality edible fungi, traditional empirical harvesting can no longer meet the requirements of large-scale and refined management of modern mushroom houses. Therefore, constructing an intelligent detection method that can evaluate the maturity of *Pleurotus ostreatus* in real time and accurately in complex environments has become a key bottleneck for the edible fungi industry to move towards digitalization and intellectualization [1].

At present, the development of *Pleurotus ostreatus* maturity detection technology can be divided into three stages. First, the traditional physicochemical detection stage: Early studies mainly relied on laboratory physicochemical analysis methods to achieve quantitative evaluation of maturity by measuring indicators such as pileus diameter, stipe length, hardness, moisture, and protein content [2,3]. However, such methods usually use offline detection, which is time-consuming and complex to operate, making it difficult to meet the needs of real-time monitoring in mushroom houses. Second, the machine vision detection stage: With the rise in machine vision technology, 2D vision systems have been used to identify the morphological characteristics of *Pleurotus ostreatus* [4]. Nevertheless, the complex scenarios in mushroom houses, such as clustered and overlapping growth of *Pleurotus ostreatus*, uneven illumination and mutual occlusion, lead to problems such as missed target detection and inaccurate feature extraction in traditional 2D vision systems, resulting in insufficient detection robustness. Third, the deep learning and multimodal perception stage: In recent years, the rapid development of deep learning technology has provided a new paradigm for the intelligent detection of edible fungi [5]. Research (e.g., Automatic mushroom harvesting robot from Mycionics in Canada) has realized automated harvesting by combining machine vision with robotic arms, providing an application paradigm for maturity detection [6]; meanwhile, classic target detection models such as Faster R-CNN and the YOLO series have laid a foundation for algorithm research and development [7,8,9]. Domestically, methods such as point cloud-based deep learning for growth monitoring, the improved OMM-YOLO model based on YOLOv5, and instance segmentation with Mask R-CNN have emerged one after another, which have significantly improved detection accuracy in complex environments [10,11,12,13].

Despite the remarkable progress made by the above technologies, existing studies still face several key challenges that directly restrict the popularization and application of the technology in actual production: First, most algorithms are mainly optimized for 2D vision or single-modal data, and fail to fully consider the extremely complex scenarios in mushroom houses such as high humidity, low light and heavy occlusion. For example, although point cloud technology can solve the occlusion problem to a certain extent, noise suppression in high-humidity environments and the cost of data acquisition remain difficult points to resolve [10,11]. Second, current studies mainly focus on the processing of RGB visible light images and lack discussion on multimodal perception technologies such as depth, infrared and multispectral technologies. In fact, such modal information has unique advantages in revealing the internal tissue structure, moisture content changes and growth stage transition of edible fungi, but it has not been fully utilized. Third, most existing studies focus on the detection of shape contours (e.g., pileus diameter and stipe length), while the systematic analysis of color changes (e.g., the color transition of pileus from yellowish green to white) and geometric features (e.g., geometric curvature and concavity–convexity of the pileus) is insufficient. These color and geometric features are often the key physiological indicators of the transition of *Pleurotus ostreatus* from the growth stage to the maturity stage. Finally, the pursuit of high-precision detection has led to a significant increase in the computational complexity of the algorithm, making it difficult to realize real-time monitoring of mushroom houses with large production capacity, and thus making them unable to meet the requirements of high throughput and high reliability on modern planting production lines.

How to construct a multimodal detection model that can both deeply explore color features and realize lightweight deployment is an urgent problem to be solved in current research. At present, there have been some explorations in academia and industry. However, most research focuses on shape and texture features, and there are relatively few studies on the separate fusion of color features. For example, YOLOv5 has been used to detect the maturity of shiitake mushrooms and HSV color space has been used to assist in judgment [14], but without deeply integrating color information into the network structure. The domestic team attempted to integrate multi-channel inputs from RGB and Lab color spaces into the Backbone of YOLOv8, which improved the recognition rate of diseased shiitake mushrooms (F1 score increased by about 3.2%) [15]. The current key technology path for color feature fusion research is: Firstly, multi-modal input fusion optimization, including a dual-branch network structure, which inputs RGB images and HSV converted images into two branches of YOLOv7 respectively, and fuses them through feature pyramid (FPN) fusion [16], combining Lab space with texture and utilizing the a/b channel (color opposition dimension) of Lab space to enhance the neck feature extraction of YOLOv6 [17]. Secondly, attention mechanism optimization mainly includes CBAM + color prior, adding the CBAM module after the SPP layer of YOLOv5, and prioritizing regions with high color saturation (S in HSV) in weight allocation [18]. Thirdly, optimizing data augmentation strategies to enhance model robustness mainly includes generating adversarial samples for color changes [19], combining adversarial samples with data augmentation [20], and generating adversarial samples in color space. The above research has achieved certain results in maturity and lesion detection, but in the recognition of mushroom maturity, it is difficult to divide the maturity at different stages of maturity interaction. There are still difficulties to overcome in the research of mushroom maturity, such as low model detection accuracy, which can easily lead to missed or misjudged maturity. Finally, the interference of natural environmental factors has not been taken into account, and the model has weak generalization ability for fuzzy and small object detection. In view of the above challenges, this study proposes a lightweight improved scheme based on color feature enhancement. We constructed a dataset of Pleurotus otreatus growth stages covering a variety of typical planting environments and an HSV hue analysis database, modelling the key hue intervals ([4°, 38°] or [110°, 155°]) of *Pleurotus ostreatus* under different environments. On this basis, we introduced an HSV hue mask as the fourth channel on the basis of the YOLOv13 base model and optimized the feature correlation calculation and attention mechanism to significantly improve maturity detection and real-time performance in complex scenarios.

## 2. Construct a Baseline Dataset for *Pleurotus ostreatus* Growth

### 2.1. Data Collection

*Pleurotus ostreatus* 969, a gray-black cultivar, was selected for the experiments in this study. The experimental cultivation sites were set at the Edible Fungi Professional Cooperative of Zhilou Village, Shilou Town, Fangshan District, Beijing, and the automated mushroom house of the Beijing Academy of Agriculture and Forestry Sciences, with the experiment periods running from 18 March to 25 March 2025 and 16 March to 25 March 2025, respectively. We tracked the entire growth cycle of *Pleurotus ostreatus* from the development of mycelia into primordia to maturity, which lasted approximately 7 days.

To ensure the quality of data collection, images were captured using a Nikon D7100 camera (Tokyo, Japan), which was fixed at a horizontal distance of 60 cm from the subjects. White balance calibration was performed with a color card to guarantee consistent light source conditions for all image acquisition. A total of 540 images were collected in the greenhouse and automated container greenhouse of the cooperative, including images of 30 mushroom spawn sticks with a complete growth cycle, captured from the front, side, top and bottom views at different growth stages. In addition, 979 images were collected in the automated mushroom house of the Beijing Academy of Agriculture and Forestry Sciences, covering multi-angle shots of 15 mushroom spawn sticks with a complete growth cycle at various developmental stages. All images were set to a resolution of 300 dpi, with a width of 6000 pixels and a height of 4000 pixels. Furthermore, an additional 1686 random images of *Pleurotus ostreatus* at all growth stages were taken. In summary, a total of 3223 images were collected for the experiments, and the detailed data distribution is presented in Table 1.

### 2.2. Data Augmentation and Preprocessing

**Figure 1:**
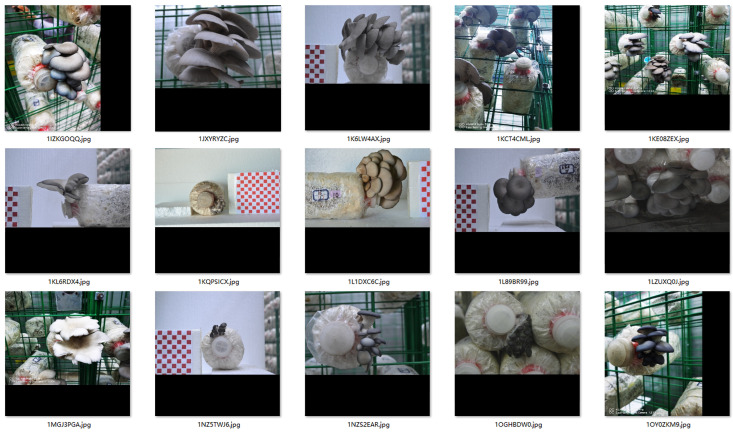
Sample data collection.

To enhance the model’s generalization capabilities in complex production scenarios, geometric deformation augmentation was applied to the collected images. This process simulates image distortions caused by variations in shooting distance and angle in real production environments, encompassing multiple deformation types including translation, rotation, and scaling. Given that this study relies primarily on color features to distinguish the maturity of *Pleurotus ostreatus*, enhancement techniques that could potentially interfere with color characteristics—such as brightness adjustment, saturation transformation, or salt-and-pepper noise addition—were avoided to preserve the original color information of *Pleurotus ostreatus*. Additionally, to meet the input size requirements of YOLO series models, all images were uniformly cropped and resized to 640 × 640 pixels. The results of the geometrically enhanced images are shown in Figure 1.

**Figure 2:**
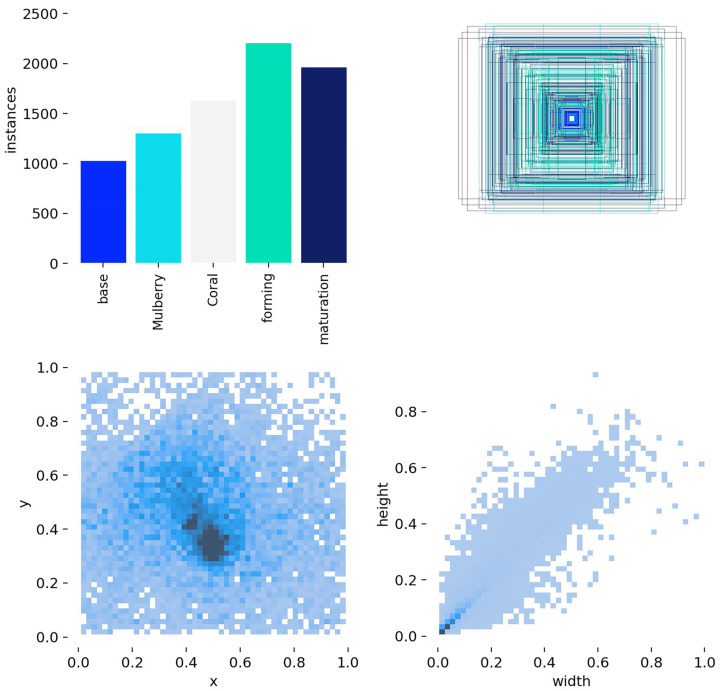
Overview of dataset classification distribution.

After merging the original captured images with the geometrically enhanced images, the total dataset size reached 4779 images. Manual annotation of the entire dataset was completed using the LabelImg 1.8.6 annotation tool; based on the morphological and color characteristics of *Pleurotus ostreatus* throughout its growth cycle, maturity was categorized into five stages: primordia stage, coral stage, young mushroom stage, formation stage, and mature stage. Annotation files adhered to the YOLO standard TXT format, ensuring each image contained at least one location and category annotation for *Pleurotus ostreatus*. The category distribution of the annotated dataset is shown in Figure 2.

Overall, the dataset covers the complete growth cycle of *Pleurotus ostreatus* from the primordia stage to the mature stage, with a total sample size of more than 8000 (refer to Table 2). The sample distribution presents a characteristic of being abundant in the middle stages and scarce at both ends, with more sufficient samples for the formation stage and mature stage. These samples provide a more abundant foundation for feature learning, enabling the model to better recognize these two key developmental stages. During model training, additional data augmentation can be performed on samples in the edge distribution to further enhance the model’s generalization capability. Finally, in accordance with the dataset division norms for object detection tasks, the combined dataset including original and augmented images was randomly split in the ratio of 8:1:1. The training set accounts for 80% and is used for the iterative learning of model parameters; the validation set accounts for 10% and is applied for hyperparameter tuning and overfitting monitoring during the training process; the test set accounts for 10% and is utilized for the independent evaluation of the model’s final performance.

## 3. Research on Environmentally Adaptive HSV Hue Modeling Mechanism

### 3.1. Construction of the Pleurotus ostreatus Color Database

The core of the *Pleurotus ostreatus* color database encompasses two major dimensions of data: cultivation environment parameters throughout the entire growth period of *Pleurotus ostreatus*, and HSV color feature data of *Pleurotus ostreatus* fruiting bodies. Cultivation environment parameters are collected in real time via specialized environmental sensors deployed within mushroom cultivation rooms. These include four key indicators affecting *Pleurotus ostreatus* growth and color variation: light intensity, carbon dioxide concentration, ambient temperature, and relative air humidity. Second, HSV color characteristics of fruiting bodies are extracted through a standardized process: High-resolution *Pleurotus ostreatus* images are first converted from RGB color space to HSV color space, which better aligns with human visual perception [21]. Color information is then decomposed into three independent components: hue, saturation, and value. Subsequently, image segmentation techniques precisely extracted the *Pleurotus ostreatus* fruiting body regions. The proportion of pixels corresponding to each HSV component relative to the total fruiting body pixels was calculated. This proportional data was stored as quantified color features, categorized by growth environment and growth stage, to construct the *Pleurotus ostreatus* HSV color analysis database. This database provides data support for subsequent correlation analysis between color features and maturity.

### 3.2. Analysis of the Hue Change Pattern of Pleurotus ostreatus

**Figure 3:**
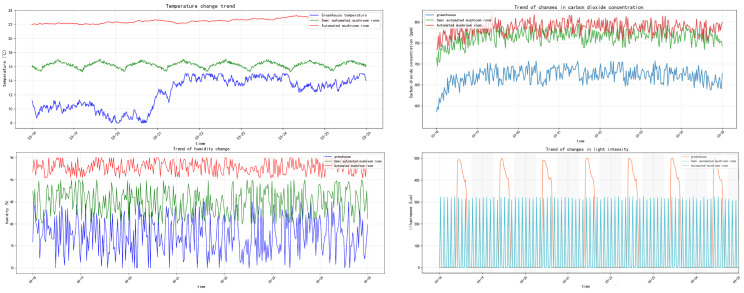
Trend chart of temperature, humidity, CO_2_ concentration, and light intensity.

**Figure 4:**
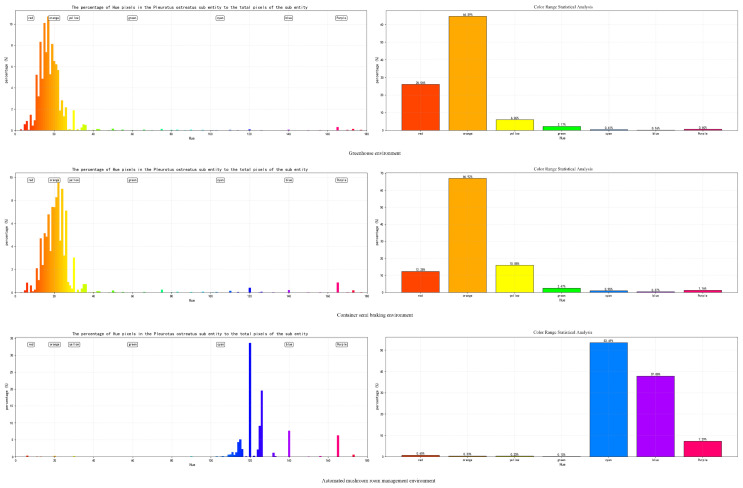
The percentage of hue pixels in the *Pleurotus ostreatus* sub-entity to the total pixels of the sub-entity.

The base color of *Pleurotus ostreatus* is genetically determined, but environmental factors such as temperature, humidity, and light intensity significantly influence the final color expression. Through in-depth analysis of the constructed *Pleurotus ostreatus* color database, despite variations in parameters like temperature, humidity, light intensity, and CO_2_ concentration across the three experimental environments (see Figure 3), *Pleurotus ostreatus* maintained relatively stable microenvironmental conditions throughout its growth period. Under controlled growth conditions, the hue changes during the growth phase of *Pleurotus ostreatus* effectively reflect its color evolution patterns. This study employs color histogram analysis for statistical evaluation. This method divides the image color space into intervals and counts the number of pixels in each interval, providing an intuitive representation of color distribution characteristics within the image [22]. Statistical analysis was performed on 3940 valid images and 10,211 annotated targets in the database; the percentage of hue pixels in *Pleurotus ostreatus* fruiting bodies relative to total pixels was calculated, yielding a color proportion bar chart for the growth period (see Figure 4).

Analysis results show:

Greenhouse environmental hue distribution (left figure): The hues of *Pleurotus ostreatus* are primarily concentrated in the 0–30° (red-orange) and 30–60° (orange-yellow) ranges, with a peak occurring in the orange region (approximately 30°). Yellow (60°) accounts for the second-highest proportion, other hues (e.g., green, blue, purple) accounted for extremely low proportions (<1% each). Color range statistics (right figure): Orange had the highest proportion (64.90%), followed by red (26.00%) and yellow (4.70%), while cool tones like blue, green, and purple all accounted for <2%. Conclusion: Under greenhouse conditions, *Pleurotus ostreatus* exhibits predominantly orange-red coloration with high color uniformity, while cool tones are virtually absent.

Semi-automated mushroom house environmental color distribution (left image): The color distribution trend resembles that of greenhouse environments, primarily concentrated in the 0–30° (red-orange) and 30–60° (orange-yellow) ranges. However, the proportion of yellow (60°) significantly increased compared to greenhouse conditions, with a small distribution appearing in the 150–180° (purple-magenta) range. Color range statistics (Right Image): Orange remains the dominant hue (64.53%), while yellow increases to 15.88%. Red decreases to 15.20%, purple accounts for 1.03%, and cool tones (blue, green) remain low (<1% each). Conclusion: Under semi-open container conditions, *Pleurotus ostreatus* coloration remains predominantly orange-yellow, but the yellow proportion increases and a small amount of purple tones appear, with color diversity slightly higher than in greenhouse conditions.

Automated mushroom house environmental color tone distribution (left chart): The color tone distribution shows significant variation: high proportion peaks occur in the 120–150° (cyan-blue) and 150–180° (blue-purple) ranges, while red (0–30°) and orange (30–60°) proportions decrease substantially. Color range statistics (right image): Cyan holds the highest proportion (39.40%), followed by blue (37.88%). Violet accounts for 7.70%, while warm tones like red, orange, and yellow each constitute <2%. Conclusion: Under automated management conditions, *Pleurotus ostreatus* predominantly exhibits cyan-blue and purple hues, with extremely low proportions of warm colors. This color profile is entirely opposite to the first two environments, indicating significantly enhanced diversity.

Analysis reveals the following: The primary hue distribution range of *Pleurotus ostreatus* exhibits significant shifts under different environmental conditions—[4°, 38°] in constant temperature and humidity environments, [4°, 38°] in semi-open greenhouses, while the fully enclosed LED environment triggers chlorophyll fluorescence due to blue light excitation, causing the hue to shift to [110°, 155°] (cyan-green to emerald-green spectrum). Based on the color hue range characteristics and environmental sensitivity patterns of *Pleurotus ostreatus*, an environment-aware hue mask generation strategy is proposed: dynamically generating a fourth-channel input that retains only pixel values within the target hue range, setting others to zero, serving as structured color prior guidance for the network. By enhancing the correlation between color features and spatial features, the model’s detection accuracy for *Pleurotus ostreatus* targets is improved. This addresses challenges in real production environments, such as complex backgrounds, dim and blurry lighting, distant detection of small targets, and difficulties in distinguishing maturity stages. The approach enhances the model’s generalization capability and detection robustness, minimizing false negatives and false positives.

## 4. Designing a Dual-Path Feature Enhancement Architecture Based on the YOLOv13 Base Model

### 4.1. Foundational Theory

Initial YOLO models were constrained by local information aggregation and pairwise correlation modeling, making it challenging to capture higher-order semantic relationships among multiple objects (e.g., occlusion and dense object scenarios). Therefore, this study employs the latest YOLOv13 version from the YOLO series. YOLOv13 was officially released on 26 June 2025, by a collaborative team from Tsinghua University, Taiyuan University of Technology, Beijing Institute of Technology, and other institutions. Its core objective is to enhance robustness in complex scene detection. As the latest iteration, its architecture incorporates the HyperACE mechanism to model higher-order visual feature correlations using hypergraphs. To optimize information flow, the FullPAD paradigm employs full-pipe feature aggregation and distribution, enhancing inter-layer synergy to improve gradient propagation and detection performance. Deep separable convolutions replace traditional large-kernel convolutions, significantly reducing model parameters and computational complexity. This design achieves many-to-many global semantic associations through hypergraph modeling, delivering notable performance advantages in small object detection and complex scenarios, providing foundational architectural support for the improvements in this paper.

Second, the HSV color space [12] decomposes color into three components: hue (H), saturation (S), and value (V). The hue component directly reflects the intrinsic characteristics of color, is less affected by light intensity, and is thus suitable as a core analytical indicator for color changes during the growth period of *Pleurotus ostreatus*.

Third, StockenAttention (stacked attention mechanism) is an optimized attention architecture that enhances the model’s ability to capture core information by constructing a multi-level cascaded attention structure. Its core approach involves leveraging cascaded iterations across multiple attention layers to progressively filter, focus on, and refine key feature information. This mechanism primarily consists of three components: a base attention module, an information stacking unit, and a gating/weight adjustment module. During operation, the first-layer attention module performs preliminary information screening on the input sequence. Subsequently, the initial attention output is fused with the original input and fed into the next-layer attention module, enabling stacked iterative attention. Each subsequent attention layer focuses on extracting critical information inadequately captured or omitted by the preceding layer, progressively refining features. Finally, a gating or weighting mechanism fuses outputs from all layers to produce a comprehensive feature representation. Its core computational logic is described by the following mathematical expressions:

Attni = Attention(Qi, Ki, Vi) Hi = Gate(Attni, Hi − 1)

where i represents the *i*-th layer of the attention module.

### 4.2. Improved YOLOv13 Model Design Incorporating Color Features

#### 4.2.1. Input Channel Expansion Optimization

**Figure 5:**
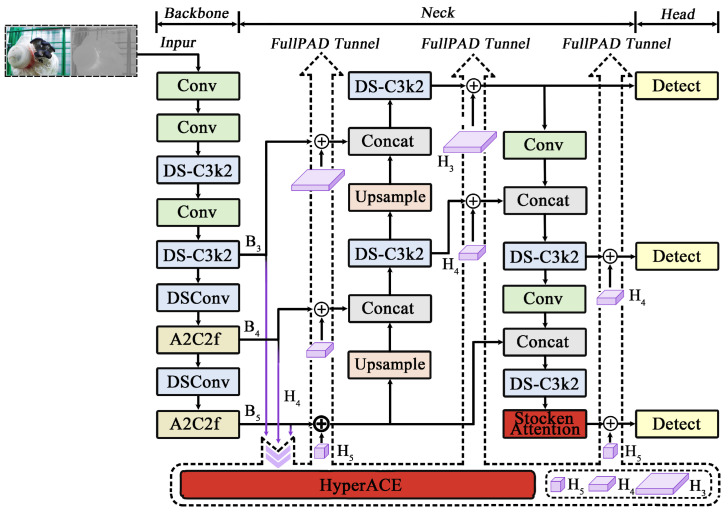
Improved YOLOv13 model design with color feature integration.

Expand the original YOLOv13’s three-channel RGB input to a four-channel input: convert the original image to HSV color space, extract the H component, then select the hue range based on the growth environment of the captured image, such as [4°, 38°] or [110°, 155°]. Pixel values outside the hue range are set to zero to generate a hue mask channel. This mask is concatenated with the RGB channels as the model’s four-channel input, enhancing the preprocessing guidance role of color semantics. The modified overall network structure is shown in Figure 5.

#### 4.2.2. ColorWeight Module Improvement: HyperACE Mechanism

**Figure 6:**
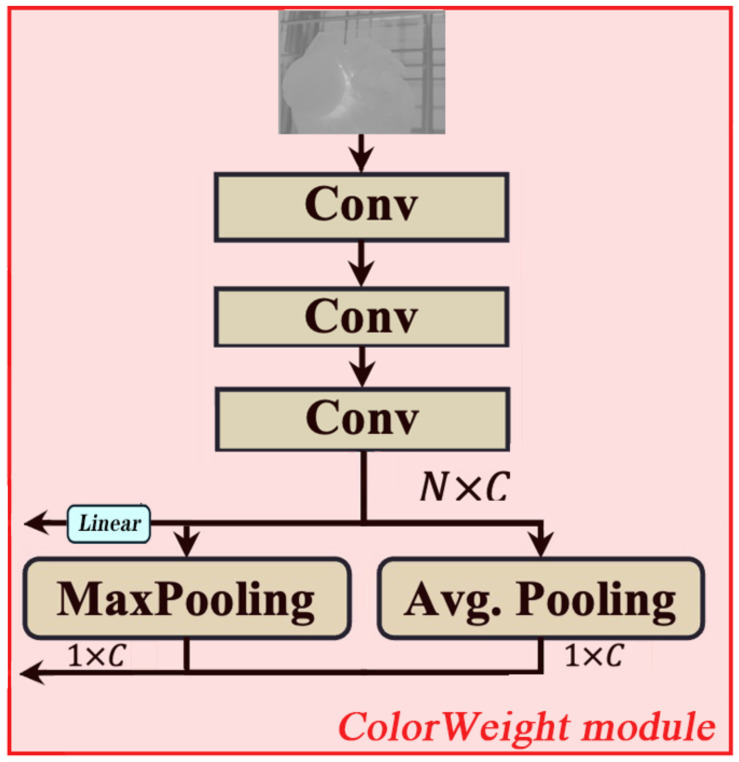
ColorWeight module.

The ColorWeight module (as shown in Figure 6) focuses on enhancing the H channel within the HSV color space based on specific hue variations observed during the growth period of *Pleurotus ostreatus*. This process aims to heighten the network’s sensitivity to target colors.

It comprises three main components: hue focusing, information statistics and fusion, and progressive feature refinement.

Hue Focusing: Through masking operations, values outside the hue ranges unrelated to *Pleurotus ostreatus* growth (e.g., [4°, 38°] and [110°, 155°]) in the image’s H channel are set to zero, directing the network’s attention toward the target color range.

Statistical Fusion: Features undergo not only linear projection but also statistical aggregation of maximum and mean values. These statistics are concatenated with original input statistics for subsequent computations. This fuses local feature responses with global statistical information, enhancing robustness.

Progressive Feature Refinement: A three-stage convolutional stack progressively reduces feature map dimensions (640 × 640 → 320 × 320 → 80 × 80 → 40 × 40) while expanding or maintaining the number of channels (1 → 32 → 64 → 64). This design mimics the visual system’s abstraction process from local details to global semantics.

Specific Implementation Flow:

**Step 1:** Input Preprocessing (Hue Optimization and Normalization)

- Purpose:

Filter irrelevant hues and standardize input.

- Operation:
  1. Create a binary mask based on the hue interval list for *Pleurotus ostreatus*: Merged = [[4°, 38°], [78, 155]].
  2. Multiply the original hue channel Xhue by the corresponding mask for its environment, setting hues outside the interval to zero.
  3. Divide the result by 180 (H channel range) to normalize to [0, 1].

**Step 2:** Three-stage Convolution Feature Extraction

- Purpose:

Gradually extract and concentrate deep features from the hue space.

- Procedure:
  1. Conv1: Fast downsampling to capture basic edges and textures. (1, 640, 640) → (32, 320, 320).
  2. Conv2: Further downsampling with increased channels to learn more complex patterns. (32, 320, 320) → (64, 80, 80).
  3. Conv3: Final downsampling to refine features. (64, 80, 80) → (64, 40, 40).

**Step 3:** Feature Linear Transformation (Projection) and Residual Connection

- Purpose: Perform spatial linear combination and information compression on extracted features.
- Operation:
  1. Flatten the 3D feature map Xhue (batch, 64, 40, 40) and convert it into a vector of (batch, 64) through global pooling to fit the nn. Linear layer.
  2. Apply a Linear (64, 64) layer to obtain pre_color_head_proj. Key Integration Point: Perform element-wise addition between pre_color_head_proj and the input from the original Adaptive Hyperedge Generation module (likely the flattened features as per Figure 2) to inject ColorWeight information.
  3. Feed the summed result into the Projection layer of the original architecture for subsequent processing.

**Step 4:** Statistical Information Enhancement

- Purpose: Utilize global statistics (maximum, mean) of features as supplementary information.
- Operation:
  1. For the final Xhue feature map, compute the global maximum and global mean along the spatial dimension, yielding two (batch, 64) vectors.
  2. Similarly, compute the global max and mean for the original preprocessed hue input.
  3. Concatenate the four statistical vectors along the feature dimension to form a (batch, 256) vector.

Project this concatenated vector through another Linear layer to output statistical features for fine-tuning color relationships.

**Figure 7:**
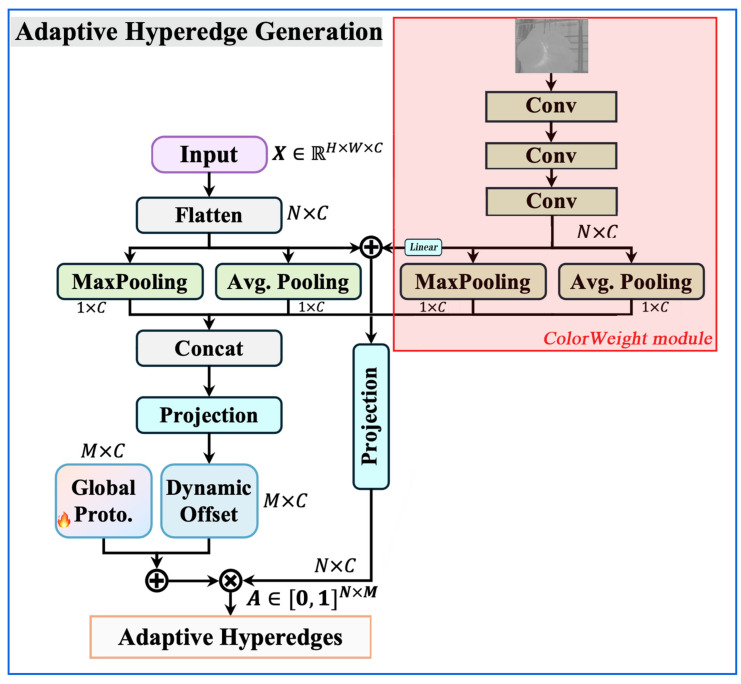
Improved adaptive hyperedge generation.

The improved HyperACE results are as follows:

Analysis of the modified Adaptive Hyperedge Generation module, as shown in Figure 7, integrates the ColorWeight module (red area on the right) into the main pipeline:

- Input:

The hue channel of the HSV image separated from the main pipeline’s input.

- Processing:

The hue channel undergoes independent processing through the complete ColorWeight module.

- Output and Fusion:
  1. Main Path Features: The pre_color_head_proj feature is added to the features from the Flatten (or equivalent) operation in the main pipeline, directly influencing subsequent Projection and Global Proto. generation.
  2. Statistical Path Features: The generated statistical feature vector may participate in the final Adaptive Hyperedge Generation via Concat or as part of the Dynamic Offset, enabling adaptive fine-tuning of color relationships.
  3. The integrated model achieves feature complementarity: the original architecture relies on MaxPooling and Avg. Pooling to obtain multi-scale statistical features, while the ColorWeight module provides domain-knowledge-based (hue) features that undergo deep processing, complementing each other. Secondly, it enhances semantic awareness: the hyperedge generation process explicitly focuses not only on spatial and texture statistics but also on color semantic information strongly correlated with the target object (*Pleurotus ostreatus*). Third, it enhances interpretability: by operating within defined hue intervals, the module makes the network’s utilization of color features partially explainable.

## 5. Model Training, Performance Evaluation, and Deployment Verification

### 5.1. Experimental Environment and Parameter Configuration

Model training was conducted on the following high-performance computing platform:

- Hardware Environment:
  1. Processor: Intel^®^ Xeon^®^ Platinum 8474C CPU (Santa Clara, CA, USA).
  2. Graphics Processor: NVIDIA GeForce RTX 3090Ti GPU (VRAM: 24 GB, Santa Clara, CA, USA).
- Software Environment:
  1. Operating System: CentOS 7.
  2. Deep Learning Framework: PyTorch 2.6.0.
  3. Computing Architecture: CUDA 12.4.
  4. Programming Language: Python 3.11.

Training parameter settings are detailed in Table 3.

### 5.2. Analysis of Training Process

**Figure 8:**
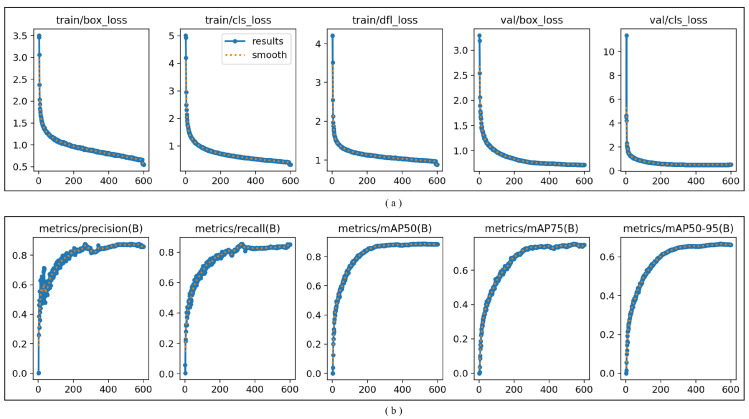
Training indicator chart. (**a**) Loss Function Analysis (**b**) Performance Metrics Analysis.

According to the training metric charts (Figure 8), the model training demonstrates a consistent convergence trend overall. Below is a detailed interpretation and comprehensive evaluation of key metrics:

#### 5.2.1. Loss Function Analysis (Figure 8a)

First, analysis of training losses (train/box_loss, train/cls_loss, train/dfi_loss) took place: All three training losses (bounding box loss, classification loss, DFI loss) rapidly decrease from higher values and gradually stabilize after 200 iterations, ultimately converging to low levels (all approaching approximately 0.5). Conclusion: The model demonstrates effective learning on the training set, with successful parameter optimization and no evident signs of overfitting or underfitting.

Second, validation loss analysis (val/box_loss, val/cls_loss) took place: The declining trend of validation loss aligns closely with training loss, ultimately stabilizing at levels comparable to training loss (no significant gap). Conclusion: The model demonstrates good generalization capability, showing neither overfitting (training loss significantly lower than validation loss) nor underfitting (validation loss persistently high).

#### 5.2.2. Performance Metrics Analysis (Figure 8b)

First, precision (metrics/precision(B)) and recall (metrics/recall(B)) analysis took place: Both metrics rapidly increase from zero, stabilizing after 200 epochs. Final precision and recall both exceed 0.8. Conclusion: The model demonstrates high accuracy in object localization and classification, achieving a balanced precision–recall tradeoff (no significant underperformance in either metric).

Second, mAP metrics analysis (mAP50, mAP75, mAP50–95) took place: mAP50 (mean average precision at IoU = 0.5): Stable above 0.8, demonstrating excellent performance. mAP75 (mean average precision at IoU = 0.75): Stable above 0.7, indicating strong detection capability for high IoU threshold targets. mAP50–95 (average precision across multiple IoU thresholds): Stable above 0.6, demonstrating good overall performance. Conclusion: The model achieves high detection performance across various IoU thresholds, particularly showing stability for medium-to-high difficulty targets (mAP75).

Comprehensive evaluation reveals the following: Good convergence: All loss functions exhibit monotonic descent and stability without fluctuations or rebounds, indicating a stable training process. Strong generalization: Minimal gap between training and validation losses, with consistent performance metrics across training and validation sets. Performance meets standards: Core metrics including precision, recall, and mAP achieve high levels. Overall model training is successful, achieving high detection performance suitable for deployment in practical scenarios.

### 5.3. Evaluation Metrics

This study employs precision (P), recall (R), and mean average precision (mAP) to evaluate model detection accuracy [23], with mAP threshold set at 0.5. Model efficiency is assessed using model parameters, computational complexity (FLOPs), and computational scale [24]. Model size is selected as the deployability evaluation metric. Frame rate per second (FPS) is chosen to assess real-time performance of model detection [25].

## 6. Experiments and Analysis

### 6.1. Performance Comparison of Models with Different Modules

To validate the necessity of each module, a controlled variable method was employed to construct comparative experiments. Using the original network as the baseline, three strategies—no module introduction, single module introduction, and combined module introduction—were applied to quantitatively analyze the independent and synergistic effects of each module on training outcomes (Table 4).

Table 5 presents the results of comparative performance tests conducted on a test set of 877 images.

Comparative Analysis of Model Performance: Using Yolov13 performance as the baseline model, its core metrics are: precision: 86.95%; recall: 87.54%; and mean average precision (mAP@0.5): 86.6%. In terms of computation and deployment costs, the model has 2.46 M parameters, requires 3.15 GFLOPs of computation, and has a model size of 5.23 MB. As a baseline model, Yolov13 strikes a balance between accuracy and efficiency, making it suitable for lightweight scenarios.

Firstly, the introduction of the attention mechanism in StokenAttention-Yolov13 has led to performance improvements—accuracy (+0.18%), recall (+1.06%), and mAP (+0.58%)—indicating that the attention mechanism enhances the ability to focus on features. Cost changes: parameters (2.72 M, +10.6%), model size (5.74 MB, +9.7%). However, the computational cost (3.15 GFLOPs) has not increased, indicating that the attention mechanism improves performance with a small number of parameters and does not significantly increase inference time. Conclusion: The attention mechanism is effective, but the improvement is limited (mAP only +0.5%).

Secondly, color features are introduced—Color-Yolov13—with performance improvements: accuracy (+1.15%), recall (+2.52%), and mAP (+2.19%). The improvement margins are significantly higher than those achieved by the attention mechanism, indicating that color features are more helpful for object detection tasks. Cost changes: The parameters (2.46 M) and model size (5.23 MB) are exactly the same as the original Yolov13, with only a slight increase in computation (3.15→3.16 GFLOPs, +0.33%). Conclusion: Color features are an extremely cost-effective improvement, significantly enhancing performance with almost no additional cost.

Finally, the joint improvement—Color-StokenAttention-Yolov13—with performance improvements in accuracy (+3.42%), recall (+3.84%), and mAP (+3.34%), achieving the best comprehensive performance, with mAP reaching 89.5%, an improvement of 2.9 percentage points over the baseline; cost changes: parameters (2.72 M), model size (5.74 MB) is the same as StokenAttention-Yolov13, and computation (3.16 GFLOPs) is only slightly higher than the original model (+0.3%). Conclusion: There is a synergistic effect between color features and attention mechanisms, and the joint improvement achieves the status of “maximizing accuracy and minimizing cost”.

The summary reveals that color features are significantly valuable: Color-Yolov13 achieves an mAP increase of 1.9% with zero additional parameter cost, demonstrating the importance of color information for target differentiation, especially in scenes with distinct color features. Secondly, the marginal benefit of the attention mechanism is as follows. Using StokenAttention alone yields limited improvement (mAP + 0.5%), but when combined with color features, the mAP further increases by 1.0%, indicating that the attention mechanism can effectively focus on key features enhanced by color. Lastly, the balance between efficiency and performance is as follows. The computational complexity (GFLOPs) of all improved models is controlled within the range of 3.15–3.16, with parameter and model size increases of ≤10%, ensuring controllable deployment costs.

### 6.2. Comparison of Mainstream Model Performances

To evaluate the performance differences between the improved model and other models in the *Pleurotus ostreatus* maturity detection task, we selected several mainstream object detection models with similar parameter counts—YOLOv12n, YOLOv11n, YOLOv10n, YOLOv9t, and YOLOv8n—for performance comparison experiments. The results are shown in Table 6.

#### 6.2.1. Detection Accuracy

The higher the mAP@0.5, the better. Among them, YOLOv9t leads with a score of 94.0%, followed by Color-StokenAttention-Yolov13 (89.5%) and YOLOv8n (88.9%). In terms of F1 score, YOLOv9t (88.49%) is close to Color-StokenAttention-Yolov13 (88.7%), indicating that both perform well in balancing precision (P) and recall (R). In terms of precision (P), YOLOv9t (92.74%) ranks the highest, followed by Color-StokenAttention-Yolov13 (89.92%), significantly outperforming lightweight models such as YOLOv10n~v12n.

#### 6.2.2. Computational Efficiency

**Frame rate (FPS, higher is better):** YOLOv8n (18.9 FPS) is slightly higher than Color-StokenAttention-Yolov13 (17.92 FPS), both of which are the fastest models in the test set; YOLOv9t has the lowest frame rate (10.17 FPS), which is only 56.8% of Color-StokenAttention-Yolov13, indicating poor real-time performance.

**Computation (FLOPs/G, lower is better):** YOLOv12n (2.99 G) and Color-StokenAttention-Yolov13 (3.16 G) have the lowest computation, making them suitable for deployment on edge devices; YOLOv9t has a computation of up to 38.78 G, which is 12.3 times that of Color-StokenAttention-Yolov13, resulting in extremely high computational cost.

**Model size (in MB, smaller is better):** Color-StokenAttention-Yolov13 (5.73 MB) is comparable to YOLOv11n (5.28 MB) and YOLOv12n (5.27 MB), making it a lightweight model.

The YOLOv9t (39 MB) model has a size 6.8 times larger than that of Color-StokenAttention-Yolov13, resulting in high deployment costs.

**Number of parameters (Parameters/M, lower is better):** YOLOv12n (2.52 M) has the fewest parameters, while Color-StokenAttention-Yolov13 (2.72 M) has slightly more, but still far less than YOLOv9t (20.16 M).

The overall analysis results are presented in Table 7

Color-StokenAttention-Yolov13: It achieves a balance between accuracy (mAP 89.5%), speed (17.92 FPS), and lightweight (5.73 MB) performance, making it suitable for most real-time detection scenarios. YOLOv9t is only recommended for high-precision requirements: if you have extremely high requirements for detection accuracy (such as scenarios sensitive to target recognition errors) and can accept high computational costs (such as server-side deployment), consider YOLOv9t. YOLOv8n is an alternative: if you require a slightly higher frame rate (18.9 FPS) and can accept a slight decrease in accuracy (mAP 88.9%), YOLOv8n is the next best choice.

### 6.3. Algorithm Verification

**Figure 9:**
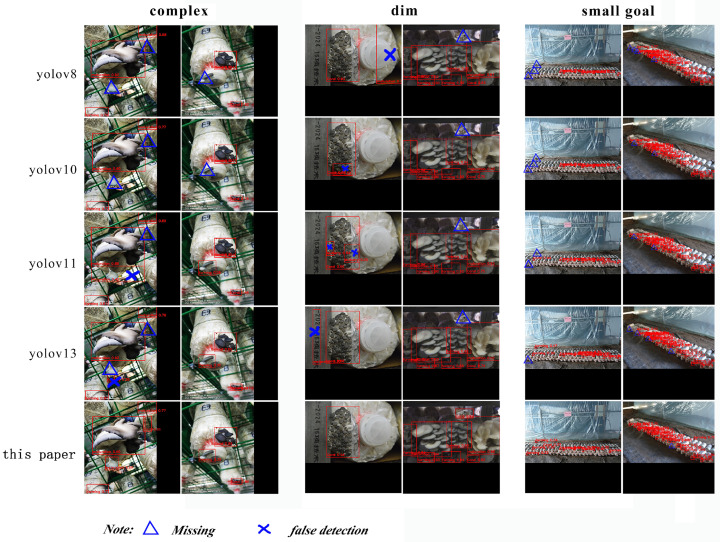
Comparison of detection results of different models under complex backgrounds, dim and blurry conditions, and small targets.

From Table 5, it can be seen that the frame rates of YOLOv9t and YOLOv12n models are relatively low, which do not meet the speed requirements for recognition. In this study, YOLOv8n, YYOLOv10n, YOLOv11n, and the improved Yolov13 algorithm were selected for visual comparison. Randomly selecting two evaluation images from three scenarios—complex background occlusion, dimness and blur, and small targets—the detection results are shown in Figure 7. Based on the experimental comparative analysis in Figure 9: firstly, in complex scenes with more interference, YOLOv8n, YOLOv10n, and YOLOv11n all exhibit missed detections, while Color-StokenAttention-Yolov13 can accurately identify them; secondly, in scenes with complex background occlusion, Color-StokenAttention-Yolov13 accurately detects the maturity of *Pleurotus ostreatus* and achieves a higher score, while the other models exhibit multiple detections and even false detections; and thirdly, in the identification of small targets such as *Pleurotus ostreatus* maturity, the scores are not high, but Color-StokenAttention-Yolov13 can identify more *Pleurotus ostreatus* targets and provide maturity identification scores. This indicates that the improved algorithm can mitigate the impact of complex background occlusion, dimness and blur, and small target environments on evaluation and recognition. The model can provide solid technical support for accurate detection of *Pleurotus ostreatus* maturity in three production environments: *Pleurotus ostreatus* greenhouses, semi-automated mushroom houses, and automated mushroom houses.

### 6.4. Analysis of Equipment Deployment Test

**Figure 10:**
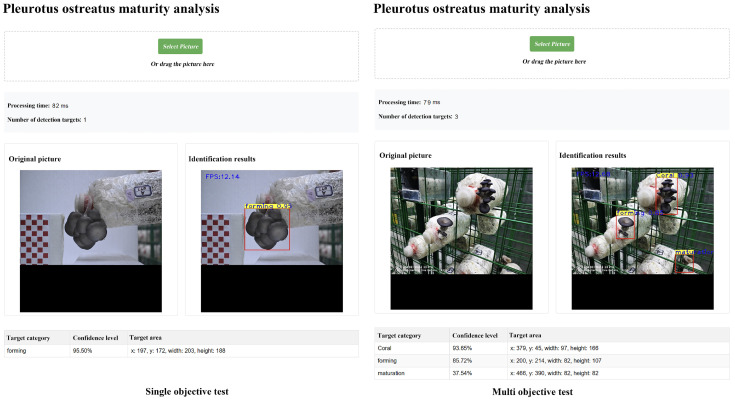
Deployment, operation, and testing results.

To verify the actual performance of the improved YOLOv13n model in a production environment, this system operates on a CentOS 7-based environment, employs a WebApi microservice architecture, and is deployed on an NVIDIA 3090Ti GPU computing environment. A 5-week closed-loop experiment was conducted in the Intelligent Mushroom House Demonstration Area of the Beijing Academy of Agriculture and Forestry Sciences, where 477 images were collected. The model achieved an F1 score of 0.91 for the maturity discrimination of *Pleurotus ostreatus*. Compared with the base model, for 692 detected objects, the number of missed detections dropped from 32 to eight, with the missed detection rate decreasing from 4.6% to 1.2%; the number of maturity misclassifications reduced from 53 to 14, representing a 73.6% decrease in the maturity misclassification rate. The test results are shown in Figure 10. Single-target processing takes only 82 ms, while multi-target processing takes 79 ms. The frame rate (FPS) reaches around 12, indicating that the model is well-designed for lightweight implementation, capable of meeting the real-time detection requirements of the mushroom house, and suitable for deployment on edge devices for online detection. The single-target test (left) was identified as forming (forming phase), with a confidence level of 95.50%, indicating that the model is highly confident in the identification of this stage. The multi-target test (right) identified three targets on the shelf of the mushroom house at once. Coral (mulberry stage): Confidence level of 93.65%, corresponding to the uppermost *Pleurotus ostreatus*, with the cap not yet differentiated and forming, presenting a coral-like cluster structure, which is an early stage of *Pleurotus ostreatus* development, and the model’s identification is accurate. Forming (forming phase): Confidence level of 85.72%, corresponding to the middle *Pleurotus ostreatus*, with the cap starting to form but not fully expanded, in a transition phase from immature to mature. Maturation (mature stage): Confidence level of 37.54%, corresponding to the lower right *Pleurotus ostreatus*, with this low confidence level indicating that the model is not confident in the judgment of this mature individual, possibly because the *Pleurotus ostreatus* is occluded or the mature characteristics are atypical. In a complex shelf scenario, the model can still distinguish between *Pleurotus ostreatus* on different mushroom spawn and identify different maturity stages, demonstrating the model’s usability in real mushroom house environments. Overall, the recognition speed is fast, the accuracy rate for early and mid-stage *Pleurotus ostreatus* is high, the positioning is precise, and it can adapt to single-target and multi-target scenarios, meeting the requirements of remote real-time detection.

## 7. Conclusions

Aiming at the core pain points in the automatic detection of *Pleurotus ostreatus*, including difficulties in multi-scale target recognition, interference from low-light environments, complex background occlusion, and challenging maturity classification, this study takes the lightweight object detection model YOLOv13n as the basic architecture and proposes a lightweight detection framework integrating physiological color prior knowledge and deep feature enhancement. This model significantly improves the adaptability to complex environments with almost no increase in parameters, computational overhead, weight file size and inference latency. The main research findings are as follows:

1. Based on three typical environments (greenhouse, semi-open mushroom shed and fully enclosed automated mushroom house) and various interference modes, 4779 *Pleurotus ostreatus* images covering five key developmental stages were collected and annotated, and a high-quality heterogeneous dataset was constructed, providing a solid data foundation for model training.
2. Based on the experimentally verified environmental hue shift law, environment-aware color priors were introduced, and a dynamic HSV hue mask generation strategy was constructed as a structured fourth-channel input, which significantly enhanced the model’s ability to distinguish target regions.
3. The color priors were innovatively combined with spatial channel attention (ColorWeight module), and “semantic consistency of same color regions” was introduced through improved hypergraph topology modeling, effectively solving the problems of sparse small target features and multi-scale target aggregation.
4. Experimental results show that with only a 10.5% increase in parameters, the mAP@0.5 is raised to 89.5% (+3.3%), the F1 score reaches 0.91, and the missed detection rate is reduced to 1.2%. Although the inference speed (13–17 FPS) is slightly lower than the traditional real-time threshold (30 FPS), it demonstrates considerable practical value owing to the 85.3% reduction in model weight and the feasibility of deployment on edge devices.

In summary, the deep integration of physiological color features of *Pleurotus ostreatus* and advanced deep learning technologies has achieved all-round performance improvements in *Pleurotus ostreatus* detection tasks, providing effective technical support for the intelligent management of smart mushroom houses.

## 8. Discussion

Although the model has achieved favorable performance in the current task of *Pleurotus ostreatus* maturity detection, there are several aspects that warrant further in-depth research and optimization. Firstly, the current model relies on the color evolution law of *Pleurotus ostreatus* throughout its growth cycle, yet the color of *Pleurotus ostreatus* varies significantly due to the notable influence of cultivar genetic characteristics and environmental factors such as temperature, humidity, and light intensity. The model thus requires sample calibration and collection for specific *Pleurotus ostreatus* cultivars, resulting in certain limitations in its scope of application. In future research, we will construct a large-scale image database of *Pleurotus ostreatus* across different cultivars and cultivation environments, conduct in-depth research on the dynamic color evolution model of *Pleurotus ostreatus* under the coupling effect of genotypes and ecological conditions, and explore universal color feature representation methods, so as to enhance the model’s transfer learning capability for unknown *Pleurotus ostreatus* cultivars. Secondly, the current detection of the model is only based on single-modal visual image data, without integrating key environmental sensing parameters such as light intensity, temperature, humidity, and CO_2_ concentration, nor incorporating temporal growth indicators including fruiting body area growth curves and growth rates. While this single-modal modeling paradigm simplifies the system structure design, it fails to comprehensively and accurately depict the evolution process of the physiological states of *Pleurotus ostreatus*, which in turn restricts the upgrading of the detection system from simple “maturity identification” to the closed-loop management mode of “growth prediction-intelligent regulation” in mushroom house production.

## Figures and Tables

**Table 1:** Data collection status table.

Category	Description		Description	
	Label Name	Image Quantity	Label Name	Image Quantity
Imaging	Complete growth cycle (greenhouse)	312	Complete growth cycle (automated house)	461
	Complete growth cycle (container)	228	Supplementary shooting (camera)	518
	Random shooting (mobile phone)	864	Random shooting (mobile phone)	840
Location	Edible Fungi Professional Cooperative of Zhilou Village, Shilou Town, Fangshan District, Beijing		Beijing Academy of Agriculture and Forestry Sciences (automated mushroom house)	
Capture Time	18 March to 25 March 2025		16 March to 25 March 2025	

**Table 2:** Dataset information.

Classes	Train	Val	Test
base	1054	243	240
mulberry	1264	264	245
coral	1694	269	312
forming	2287	382	434
maturation	1920	306	365

**Table 3:** Training setting parameters.

Training Parameter	Value
Batch size	16
Epochs	600
Learn rate	0.01
Momentum	0.937
Weight decay times	0.0005

**Table 4:** Specific experimental conditions.

Experimental Group	Module Configuration	Core variable Difference	Verification Goal
Control group	No network modification	No module intervention	Baseline performance
Experimental Group 1	Only added the StokenAttention	Single module	Functions independently
Experimental Group 2	Only add HSV-H layer	4-channel input	Single input added
Experimental Group 3	Simultaneous addition of HSV-H layer + Color module	Dual-module combination (feature enhancement + color constraint)	Multi-module synergistic effect

**Table 5:** Comparison of model detection performance introduced by different modules.

Models	P/%	R/%	mAP@0.5/%	Param./M	FLOPs/G	Size/MB
Yolov13n	86.95	87.54	86.6	2.46	3.15	5.23
StokenAttention- Yolov13	87.11	88.47	87.1	2.72	3.15	5.74
Color- Yolov13	87.95	89.75	88.5	2.46	3.16	5.23
This Paper	89.92	90.91	89.5	2.72	3.16	5.73

**Table 6:** Performance comparison table of mainstream models.

Models	P/%	R/%	F1 Score	mAP@0.5/%	Param./M	FLOPs/G	Size/MB	Fps
YOLOv12n	83.95	88.54	79.14	85.6	2.52	2.99	5.27	10.15
YOLOv11n	86.41	89.63	81.53	89.1	2.59	3.22	5.28	13.39
YOLOv10n	87.14	89.10	82.37	88.4	2.71	4.20	5.59	14.04
YOLOv9t	92.74	96.11	88.49	94.0	20.16	38.78	39	10.17
YOLOv8n	84.75	88.76	81.04	88.9	3.01	4.1	6.03	18.9
This Paper	89.92	90.91	88.7	89.5	2.72	3.16	5.73	17.92

**Table 7:** Horizontal comparison of models.

Model	Advantages Scenario	Core Bottleneck
YOLOv10n~v12n	Extremely lightweight	With relatively low accuracy (mAP < 89%)
YOLOv9t	High-precision detection	Large computation, slow speed, and bulky model
YOLOv8n	Medium accuracy + high speed	Its accuracy is lower than that of Color-StokenAttention-Yolov13
This Paper	Lightweight deployment, high real-time performance requirements	Slightly lower accuracy than YOLOv9t

## Data Availability

The original contributions presented in this study are included in the article. Further inquiries can be directed to the corresponding author.

## References

[1] Shi L., Bai Z., Yin X., Wei Z., You H., Liu S., Wang F., Qi X., Yu H., Bi C. (2025). OMB-YOLO-tiny: A Lightweight Detection Model for Damaged *Pleurotus ostreatus* Based on Enhanced YOLOv8n. Horticulturae.

[2] Jonathan S.G., Esho E.O., Ajayi I.A. (2011). Chemical compositions of oyster mushrooms (*Pleurotus ostreatus* and *Pleurotus pulmonarius*) under storage. Nat. Prod..

[3] Golian M., Chlebová Z., Žiarovská J., Benzová L., Urbanová L., Hovaňáková L., Chlebo P., Urminská D. (2022). Analysis of Biochemical and Genetic Variability of *Pleurotus ostreatus* Based on the β-Glucans and CDDP Markers. J. Fungi.

[4] Lee C.-H., Choi D., Pecchia J., He L., Heinemann P. (2019). Development of A Mushroom Harvesting Assistance System Using Computer Vision.

[5] Nuwayhid S., Charisis C., Argyropoulos D. Novel Mask R-CNN Based Mushroom Cluster Tracking in Time-Lapse Images from a Farm Environment. Proceedings of the 2024 IEEE 2nd Conference on AgriFood Electronics (CAFE).

[6] Horticulture New Knowledge Official Account Canada: Mushroom Picking Robots Put into Use. 2025, Volume 5, p. 125. https://d.wanfangdata.com.cn/periodical/zgsyj202505022.

[7] Ziliang Z., Yingzhou Y., Zhuo B. Identification Method of Pleurotus Eryngii Cut Part Based on YOLO V5s. Proceedings of the 2023 3rd International Conference on Computer, Control and Robotics (ICCCR).

[8] Wu J., Wu C., Guo H., Bai T., He Y., Li X. (2023). Research on Red Jujubes Recognition Based on a Convolutional Neural Network. Appl. Sci..

[9] Retsinas G., Efthymiou N., Anagnostopoulou D., Maragos P. (2023). Mushroom Detection and Three Dimensional Pose Estimation from Multi-View Point Clouds. Sensors.

[10] Sujatanagarjuna A., Kia S., Briechle D.F., Leiding B. (2023). MushR: A Smart, Automated, and Scalable Indoor Harvesting System for Gourmet Mushrooms. Agriculture.

[11] Retsinas G., Efthymiou N., Maragos P. Mushroom Segmentation and 3D Pose Estimation from Point Clouds using Fully Convolutional Geometric Features and Implicit Pose Encoding. Proceedings of the 2023 IEEE/CVF Conference on Computer Vision and Pattern Recognition (CVPR).

[12] Wang Y., Yang L., Chen H., Hussain A., Ma C., Al-Gabri M. (2022). Mushroom-YOLO: A deep learning algorithm for mushroom growth recognition based on improved YOLOv5 in agriculture 4.0. Proceedings of the 2022 IEEE 20th International Conference on Industrial Informatics (INDIN) 2022, Perth, Australia, 25–28 July 2022.

[13] Zhu F., Sun Y., Zhang Y., Zhang W., Qi J. (2023). An Improved MobileNetV3 Mushroom Quality Classification Model Using Images with Complex Backgrounds. Agronomy.

[14] Wang L., Wang B., Li D., Zhao Y., Wang C., Zhang D. (2023). Object detection and classification of *Pleurotus ostreatus* using improved YOLOv5. Trans. Chin. Soc. Agric. Eng..

[15] Boondach M., Chaisiriprasert P. (2024). Improving Durian Leaf Disease Detection Using LAB Color Space and CLAHE Technique with YOLOv8 Integration. Proceedings of the 2024 8th International Conference on Information Technology (InCIT), Chonburi, Thailand, 14–15 November 2024.

[16] Wang X., Wu Z., Xiao G., Han C., Fang C. (2024). YOLOv7-DWS: Tea bud recognition and detection network in multi-density environment via improved YOLOv7. Front. Plant Sci..

[17] Shi Y., Chen Y., Huang X., Wang Z., Yu W., Fang Y. (2026). DST-Net: A Dual-Stream Transformer with Illumination-Independent Feature Guidance and Multi-Scale Spatial Convolution for Low-Light Image Enhancement. arXiv.

[18] Woo S., Park J., Lee J.-Y., Kweon I.S. (2018). CBAM: Convolutional Block Attention Module. ECCV 2018. https://arxiv.org/pdf/1807.06521.

[19] Wang R., Huang Z., Chen Z., Liu L., Chen J., Wang L. Anti-Forgery: Towards a Stealthy and Robust DeepFake Disruption Attack via Adversarial Perceptual-aware Perturbations. Proceedings of the International Joint Conference on Artificial Intelligence (IJCAI).

[20] Volpi R., Namkoong H., Sener O., Duchi J.C., Murino V., Savarese S. (2018). Generalizing to Unseen Domains via Adversarial Data Augmentation. Adv. Neural Inf. Process. Syst. (NeurIPS).

[21] Narkhede P.R., Gokhale A.V. (2015). Color Image Segmentation using Edge Detection and Seeded Region Growing Approach for CIELab and HSV Color Spaces. Proceedings of the 2015 International Conference on Industrial Instrumentation and Control (ICIC), Pune, India, 28–30 May 2015.

[22] Rimiru R.M., Gateri J., Kimwele M.W. (2022). GaborNet: Investigating the importance of color space, scale and orientation for image classification. PeerJ Comput. Sci..

[23] Yang Z., He X., Gao J., Deng L., Smola A. (2016). Stacked Attention Networks for Image Question Answering. Proceedings of the IEEE Conference on Computer Vision and Pattern Recognition (CVPR), Las Vegas, NV, USA, 27–30 June 2016.

[24] Everingham M., Van Gool L., Williams C.K.I., Winn J., Zisserman A. (2010). The PASCAL Visual Object Classes (VOC) Challenge. Int. J. Comput. Vis. (IJCV).

[25] Howard A.G., Zhu M., Chen B., Kalenichenko D., Wang W., Weyand T., Andreetto M., Adam H. (2017). MobileNets: Efficient Convolutional Neural Networks for Mobile Vision Applications. arXiv.

